# Long-term tolerability and effectiveness of raltegravir in Japanese patients: Results from post-marketing surveillance

**DOI:** 10.1371/journal.pone.0210384

**Published:** 2019-01-09

**Authors:** Naho Kuroishi, Asuka Watananbe, Ryuta Sakuma, Daniel J. Ruzicka, Mitsuyoshi Hara

**Affiliations:** 1 Medical Affairs MSD K.K., Tokyo, Japan; 2 Pharmacovigilance Area MSD K.K., Tokyo, Japan; Consejo Superior de Investigaciones Cientificas, SPAIN

## Abstract

Antiretroviral agents are approved in Japan based on non-clinical and clinical data reported from overseas. Neither the long-term tolerability nor the effectiveness of raltegravir or other integrase strand transfer inhibitors in Japan is known. This study reports on the long-term tolerability and effectiveness of raltegravir in Japanese clinical practice using data collected through approximately 9 years of post-marketing surveillance. This observational survey used data on human immunodeficiency virus (HIV) infected patients initiated treatment with raltegravir between 2008 and 2017 in the HIV-related drug (HRD) cooperative survey to assess the safety and effectiveness of raltegravir in real world clinical practice. There were totally 1,303 patients prescribed raltegravir across 30 institutions; 1,293 patients and 1,178 patients were included for the safety and effectiveness analyses, respectively. The overall risk of adverse drug reaction was 17.25%, with abnormal hepatic function and hyperlipidaemia (<1.5%) having the highest proportion. Median HIV-1 RNA viral loads rapidly decreased below 40 copies/mL after 3 months of raltegravir use in treatment-naïve patients, and consistently sustained below 40 copies/mL after the start of raltegravir use in treatment-experienced patients. Among the patients who were treated for 7 years, 92.00% (95% CI: 73.97–99.02) maintained HIV-1 RNA viral load below 50 copies/mL. Additionally, CD4^+^ cell counts exceeded >500 cells/μL in treatment-naïve and treatment-experienced patients after 3 years and 4 years of treatment, respectively. In Japanese HIV patients, long-term treatment with raltegravir is well-tolerated and effective at viral suppression as measured by HIV-1 RNA levels and subsequent change in CD4^+^ cell counts. Such benefits can be expected for not only treatment-naïve but also treatment-experienced patients.

## Introduction

Antiretroviral therapy (ART) has dramatically improved the overall survival of people living with human immunodeficiency virus (HIV), but the life-long tolerability of ART has yet to be revealed. Raltegravir (Isentress, Merck & Co., Inc., Kenilworth, NJ, USA) is the world’s first in class of HIV integrase strand transfer inhibitor (INSTI) for the treatment of HIV-1 infection. The safety and efficacy of raltegravir administration in clinical trials for up to 5 years among HIV-infected patients in North American, South American, Australian, and South Asian countries have been previously reported [1–8]. After its approval in 2007 in the United States, 112 other countries also approved raltegravir [9]. In Japan, use of a 400-mg raltegravir tablet twice daily was approved by the regulatory authority in June 2008, and this drug has been marketed in Japan since July 2008. However, the safety and efficacy of raltegravir has not been evaluated in clinical trials specifically targeting Japanese patients.

In Japan, around 1,500 HIV/Acquired immunodeficiency syndrome (AIDS) cases have been reported annually since 2007 and 1,448 HIV/AIDS (1,380 males) cases were reported in 2016. Men who have sex with men (MSM), including bisexual contacts, made up 73% (735/1,011) of all HIV cases and the majority was in their 20’s to 40’s. The cumulative number of reported HIV/AIDS cases (excluding coagulating agent-related cases) was 27,443 as of the end of 2016 [10].

A rapid drug approval process is applied for antiretroviral agents in Japan, and this approval is based on non-clinical and clinical data reported from overseas as an orphan drug. As such, raltegravir was approved for clinical use under the condition of conducting a post-marketing surveillance to collect domestic data on the safety and effectiveness of this agent in clinical practice. Because antiretroviral agents are usually used concomitantly with other anti-HIV drugs, all pharmaceutical companies with an approved anti-HIV drug work together to conduct a HIV-related drug (HRD) cooperative survey [11].

The surveillance of raltegravir through the HRD cooperative survey started at the time of its marketing, in July 2008. To date, no data on the long-term tolerability and effectiveness in Japan of either raltegravir or other INSTI agents are available. Furthermore, only a few studies on the long-term effectiveness of key drug have been conducted in other countries; they include the 12-year experience of nevirapine use in France [12], a 9-year cohort of efavirenz and nevirapine in Senegal [13], a 10-year cohort on efavirenz and nevirapine in Uganda [14], and the 7-year efficacy of a lopinavir/ritonavir-based regimen in the United States [15]. Therefore, the objective of the present study was to assess the long-term tolerability and effectiveness of raltegravir in Japanese clinical practice, using the HRD cooperative survey data collected as post-marketing surveillance over the approximately 9 years from 2008 to 2017.

## Methods

### Study design

This observational survey used post-marketing surveillance data collected for the HRD cooperative survey and was conducted in accordance with the requirements of the pharmaceutical affairs law and the ministerial ordinance of ‘Good Post-Marketing Study Practice (GPSP)’. At their discretion, investigators of the contracted institutions registered patients who were newly prescribed with particular antiretroviral agents and provided data on the safety and effectiveness of the antiretroviral agents for each treated patient. Patient background information, such as age, sex, comorbidity on administration (including hepatic disease and renal disease), presence of allergy, status of anti-HIV drug use, and concomitant drug use (dose level, dosing period), was collected at registration. Data were collected by filling out a case report form upon registration and annually thereafter. If a patient experienced an adverse event (AE), the corresponding data were reported immediately using the AE report form. The management of the HRD cooperative survey was delegated to CMIC PMS Co., Ltd. (Tokyo, Japan). All data were fully anonymized before the authors of this study accessed them.

### Study population

Data on patients registered as newly treated with raltegravir between July 2008 (the beginning of raltegravir marketing) and March 2017 were drawn from the HRD cooperative survey for this study. Of the 33 institutions in which patients were prescribed raltegravir, three institutions did not agree to the disclosure of information, so they were excluded from these analyses.

### Ethics statement

This post-marketing surveillance (PMS) conducted in accordance with Good Post-marketing Study Practice for drugs [GPSP; Ministry of Health, Labour, and Welfare (MHLW) ministerial ordinance]. The survey protocol was reviewed and approved in advance by the Pharmaceuticals and Medical Devices Agency (PMDA). The requirements of ethical approval by an institutional review board and informed consent from individual patients for this survey were waived. However, all 30 facilities obtained approval in advance by their institutional review board according to each institutional rule. Names of the 30 facilities were anonymized for publication due to the contract of HRD cooperative survey. Of these 30 facilities, 29 are HIV specialized hospitals, 22 are national or public hospitals, and mean number of beds are 758 (range 135–1,379).

### Safety assessments

To assess the safety of raltegravir, AEs and abnormal variations in laboratory test parameters (i.e. haematology test, clinical biochemical test, urine analysis, and ocular abnormalities) were collected during the treatment period. All events with a relationship between the AE and drug were defined as adverse drug reactions (ADRs). AEs and ADRs were classified according to the Medical Dictionary for Regulatory Activities/Japanese version (MedDRA/J Ver.20.0).

### Effectiveness assessments

For the assessment of effectiveness, the CD4^+^ cell counts, HIV-1 RNA levels, and HIV infection clinical stages (i.e. Centers for Disease Control and Prevention [CDC] categories [16]) before and after raltegravir administration (baseline and treatment period) were collected. Infection clinical stages were classified as follows: category A was defined as asymptomatic HIV infection, category B was defined as symptomatic HIV infection, and category C was defined as acquired immunodeficiency syndrome (AIDS).

### Statistical analysis

Patient demographics, clinical characteristics, AEs, and ADRs were summarized descriptively from the collected data. The long-term safety of raltegravir was assessed by summarizing the risk of ADRs by the time of occurrence from the start of raltegravir treatment: <90 days, between 90 and <180 days, between 180 and <270 days, between 270 days and <1 year, between 1 year and <2 years, between 2 and <3 years, between 3 and <4 years, between 4 and <5 years, between 5 and <6 years, and ≥6 years. To assess potential risk factors for ADRs, the ADR occurrence was further analysed according to baseline characteristics using a Fisher’s exact test or Cochran-Armitage test. Changes in the median HIV-1 RNA levels and median CD4^+^ cell counts were calculated over time at 0, 3, 6, 12, 24, 36, 48, 60, 72, 84, and 96 months after the start of raltegravir treatment for treatment-naïve patients and treatment-experienced patients. The proportion of patients with <50 copies/mL of HIV-1 RNA, including blip cases (i.e. patients with <50 copies/mL of HIV-1 RNA with transient increases up to 1,000 copies/mL), was also calculated for the virologic assessment, and its 95% confidential intervals was estimated using Clopper-Pearson method. All statistical tests were two-sided at a significance level of 0.05 and were conducted using SAS Release 9.4 (SAS Institute, Inc., Cary, NC, USA).

## Results

### Patient disposition

**Fig 1:**
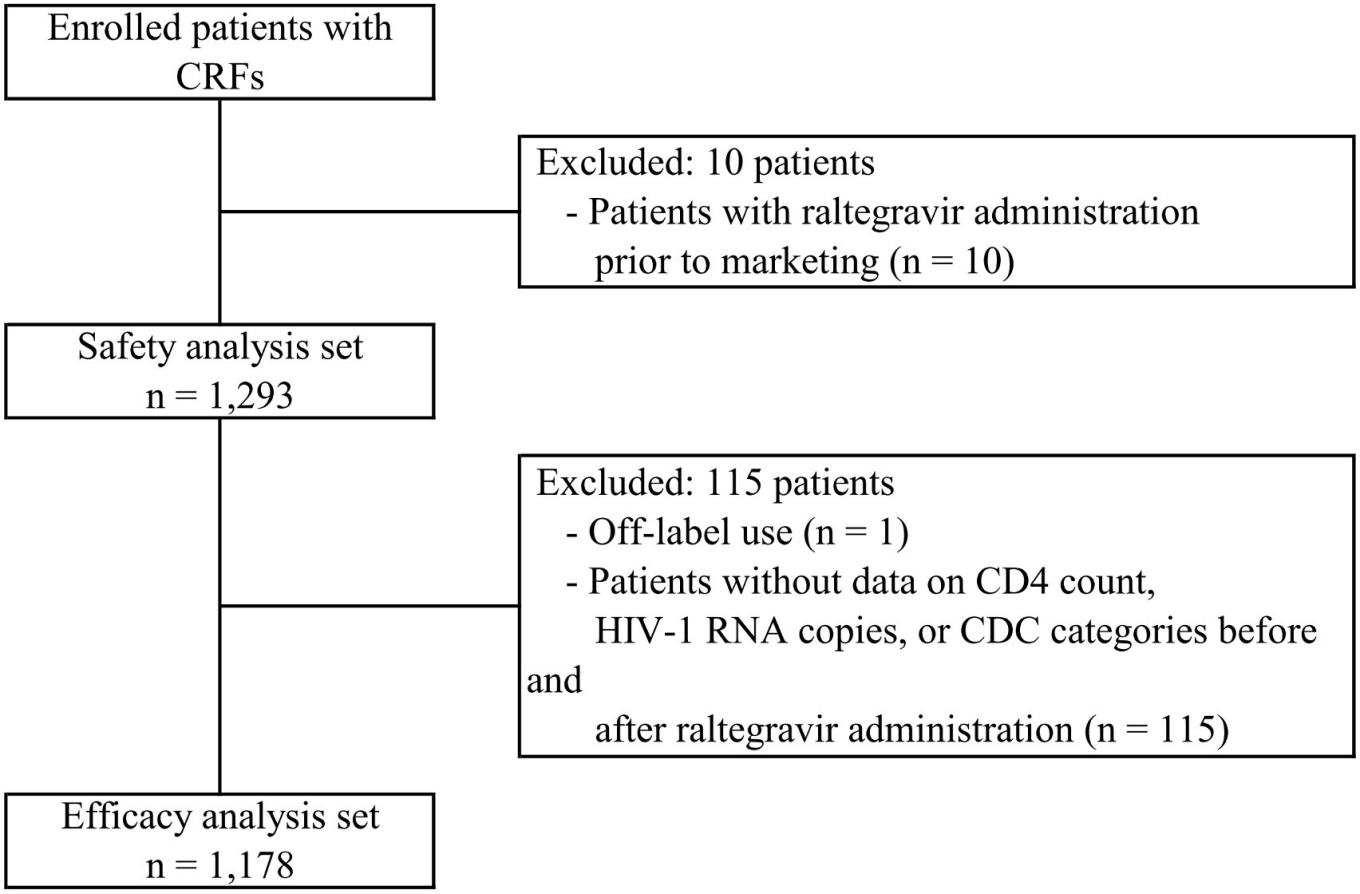
Patient disposition. CRF: case report form, CDC: Centers for Disease Control and Prevention.

A total of 1,303 patients were prescribed with raltegravir across 30 institutions of the HRD cooperative survey. Of these, 10 patients were excluded due to the fact that their prescription of raltegravir was written before its launch in Japan. 1,293 patients were included for the safety analysis. For the analysis of raltegravir effectiveness, one patient with an off-label use of raltegravir and 115 patients with no recorded CD4^+^ cell counts, number of HIV-1 RNA copies, or CDC categories either before or after raltegravir administration were excluded. Finally, data from the remaining 1,178 patients were included for the effectiveness analysis (Fig 1).

### Patient demographics and clinical characteristics

The patient demographics and clinical characteristics at baseline are summarized in Table 1. In the safety analysis set (n = 1,293), 94.1% of patients were male, and the median age at baseline was 41 years (Interquartile range, IQR: 34–50 years). The majority of patients belonged to pre-treatment CDC category A (54.5%), followed by categories C (30.9%) and B (6.0%). The prevalence of comorbidity was 68.5%. Hepatic disease and renal disease were present at baseline in 25.1% and 4.9% of the patients, respectively. Comorbidities that were reported in >50 patients at baseline were: pneumocystis jirovecii pneumonia (n = 122; 9.4%), hypertension (n = 99; 7.7%), hyperlipidaemia (n = 68; 5.3%), insomnia (n = 66; 5.1%), diabetes mellitus (n = 55; 4.3%), and hyperuricaemia (n = 50; 3.9%). Most patients (99.1%) were prescribed with a dose of 800 mg of raltegravir per day. The median treatment period from the start of raltegravir administration was 1,123 days, and 84.2% of the patients had been treated with raltegravir longer than 1 year.

**Table 1:** Patient baseline characteristics.

Characteristics	Total	
	(n = 1,293)	
Age (Years) median	41.00 (IQR: 34–50)	
≤14 years	0	(0.0)
15‒<65 years	1,218	(94.2)
≥65 years	75	(5.8)
Sex		
Male	1,217	(94.1)
Clinical status of HIV infection		
(CDC category) prior to raltegravir administration for patients aged ≥15^a^		
A	705	(54.5)
B	77	(6.0)
C	399	(30.9)
Unknown/not stated	112	(8.7)
Comorbidity, present	886	(68.5)
Hepatic disease ^b^, present	325	(25.1)
Hepatitis A	3	(0.2)
Hepatitis B	130	(10.1)
Hepatitis C	115	(8.9)
Others	64	(4.9)
Renal disease ^c^, present	63	(4.9)
Treatment period with raltegravir		
<90 days	63	(4.9)
90‒<180 days	40	(3.1)
180‒<270 days	56	(4.3)
270‒<1 year	43	(3.3)
1‒<2 years	196	(15.2)
2‒<3 years	229	(17.7)
3‒<4 years	248	(19.2)
4‒<5 years	199	(15.4)
5‒<6 years	141	(10.9)
≥6 years	76	(5.9)

^a^CDC category A was defined as asymptomatic HIV infection, category B was defined as symptomatic HIV infection, and category C was defined as acquired immunodeficiency syndrome (AIDS).

^b^Hepatic disease was defined if checkbox with “Hepatitis A, B, C, or other hepatic disorders” was selected as present, or hepatic related disease [Preferred Term (PT)/Lowest Level Terms (LLT) linked Standarized MedDRA Queries (SMQ) of “Hepatic disorders”] was reported in other comorbidity column.

^c^Renal disease was defined if checkbox with “Renal disorders” was selected as present, or renal related disease [primary System Organ Class (SOC) of “Renal and urinary disorders” and PT/LLT linked SMQ of “Acute renal failure” or “Renovascular disorders”] was reported in other comorbidity column.

Note: Data are presented as frequencies and percentages unless otherwise indicated. Due to rounding, some percentages may not sum up to 100%.

### Safety analysis

#### Adverse drug reactions and laboratory abnormalities

Of 1,293 patients in the safety analysis population, 346 ADRs were reported in 223 patients, with an overall risk rate of 17.25%. Of those, 55 serious ADRs were reported in 31 patients (2.40%). The most common ADRs were abnormal hepatic function (17 events; 1.31%), hyperlipidaemia (14 events; 1.08%), renal impairment (11 events; 0.85%), hyperuricaemia (11 events; 0.85%), liver disorder (11 events; 0.85%), dyslipidaemia (9 events; 0.70%), hypertension (9 events; 0.70%), hypertriglyceridaemia (8 events; 0.62%), immune reconstitution inflammatory syndrome (8 events; 0.62%), insomnia (7 events; 0.54%), and increased blood creatinine levels (7 events; 0.54%) (Table 2).

**Table 2:** Adverse drug reactions and laboratory abnormalities reported in patients with ≥ 5 events (n = 1,293).

Adverse drug reactions (MedDRA system organ class and term)	Number of event	(%)
Infections and infestations (n = 11, 0.85%)		
Neoplasms benign, malignant and unspecified (including cysts and polyps) (n = 6, 0.46%)		
Blood and lymphatic system disorders (n = 6, 0.46%)		
Immune system disorders (n = 8, 0.62%)		
Immune reconstitution inflammatory syndrome	8	(0.62)
Endocrine disorders (n = 1, 0.08%)		
Metabolism and nutrition disorders (n = 53, 4.10%)		
Diabetes mellitus	5	(0.39)
Hypertriglyceridaemia	8	(0.62)
Hyperuricaemia	11	(0.85)
Dyslipidaemia	9	(0.70)
Hyperlipidaemia	14	(1.08)
Psychiatric disorders (n = 13, 1.01%)		
Insomnia	7	(0.54)
Nervous system disorders (n = 13, 1.01%)		
Headache	5	(0.39)
Eye disorders (n = 1, 0.08%)		
Cardiac disorders (n = 6, 0.46%)		
Vascular disorders (n = 11, 0.85%)		
Hypertension	9	(0.70)
Respiratory, thoracic and mediastinal disorders (n = 4, 0.31%)		
Gastrointestinal disorders (n = 25, 1.93%)		
Hepatobiliary disorders (n = 36, 2.78%)		
Hepatic function abnormal	17	(1.31)
Liver disorder	11	(0.85)
Skin and subcutaneous tissue disorders (n = 19, 1.47%)		
Musculoskeletal and connective tissue disorders (n = 11, 0.85%)		
Renal and urinary disorders (n = 19, 1.47%)		
Renal impairment	11	(0.85)
Reproductive system and breast disorders (n = 1, 0.08%)		
General disorders and administration site conditions (n = 8, 0.62%)		
Investigations (n = 55, 4.25%)		
Blood creatinine increased	7	(0.54)
Gamma-glutamyltransferase increased	5	(0.39)
Renal function test abnormal	5	(0.39)
Injury, poisoning and procedural complications (n = 4, 0.31%)		

For predefined events of interest on monitoring the safety of raltegravir, the most frequently reported ADRs that were related to musculoskeletal and connective tissue disorders were back pain (3 events; 0.23%), followed by rhabdomyolysis (2 events; 0.15%) and osteoporosis (2 events; 0.15%) (Table 2, S1 Appendix). As for skin and subcutaneous tissue disorders, pruritus (4 events; 0.31%) and rash (3 events; 0.23%) were reported. The reported cardiac disorder-related ADRs were cardiac failure (2 events; 0.15%) and atrial fibrillation, myocardial infarction, supraventricular extrasystoles, and ventricular extrasystoles (1 event each; 0.08%). Benign, malignant, and unspecified neoplasms were reported as ADRs in 6 cases (0.46%), including Kaposi’s sarcoma (2 events; 0.15%), bladder cancer (1 event; 0.08%), skin papilloma (1 event; 0.08%), squamous cell carcinoma of the lung (1 event; 0.08%), and thymoma (1 event; 0.08%).

#### Long-term safety

The ADR risk by the time period from raltegravir treatment initiation was 10.20% (20/196 cases), 16.16% (37/229 cases), 16.13% (40/248 cases), 22.11% (44/199 cases), 18.44% (26/141 cases), and 23.68% (18/76 cases) for treatment durations of between 1 and <2 years, 2 and <3 years, 3 and <4 years, 4 and <5 years, 5 and <6 years, and ≥6 years, respectively (Table 3). In particular, the risk of metabolism and nutrition disorders increased as the raltegravir treatment period lengthened: 1.02% (2/196 cases), 2.18% (5/229 cases), 4.44% (11/248 cases), 7.04% (14/199 cases), 7.09% (10/141 cases), and 10.53% (8/76 cases) after treatment periods of between 1 and <2 years, 2 and <3 years, 3 and <4 years, 4 and <5 years, 5 and <6 years, and ≥6 years, respectively (Cochrane-Armitage test: *p* < 0.001).

**Table 3:** Risk of adverse drug reactions by time period from the start of raltegravir treatment.

	<90 days	90‒<180 days	180‒<270 days	270 days‒<1 year	1‒<2 years	2‒<3 years	3‒<4 years	4‒<5 years	5‒<6 years	≥6 years	unknown/not stated	Total
**Patients, n**	63	40	56	43	196	229	248	199	141	76	2	1,293
**Patients with ADR, n**	17	8	9	3	20	37	40	44	26	18	1	223
**ADR, event**	25	12	16	3	25	56	65	70	42	30	2	346
**Patients with ADR,%**	26.98	20.00	16.07	6.98	10.20	16.16	16.13	22.11	18.44	23.68	50.00	17.25

ADR: adverse drug reactions

#### Assessment of baseline factors affecting ADR development

ADR rates were significantly higher in patients with an older age (*p* = 0.039), CDC category of B (*p* = 0.019), the presence of any comorbidities (19.86%, *p* < 0.001), the presence of hepatic disease (22.77%, *p* = 0.003), the presence of allergic disease (26.49%, *p* < 0.001), or an increasing number of concomitant medications (*p* < 0.001) (Table 4).

**Table 4:** Risk of adverse drug reactions according to baseline characteristics.

Patient characteristics	n	Adverse drug reactions			p value^a^
		n	Number of events	Proportions of patients with ADR	
**Total**	1,293	223	346	17.2	-
**Sex**					
**Male**	1,217	209	321	17.17	0.755
**Female**	76	14	25	18.42	
**Age**					
**≤14 years**	0	0	0	-	0.039
**15‒<25 years**	48	6	10	12.50	
**25‒<35 years**	282	40	57	14.18	
**35‒<45 years**	473	83	119	17.55	
**45‒<55 years**	258	46	72	17.83	
**55‒<65 years**	157	33	66	21.02	
**65‒<75 years**	67	12	17	17.91	
**≥75 years**	8	3	5	37.50	
**Pre-treatment CDC category (age ≥15)**					
**A**	705	106	162	15.04	0.019
**B**	77	18	23	23.38	
**C**	399	83	138	20.80	
**Presence of comorbidities**					
**Present**	886	176	284	19.86	<0.001
**Absent**	407	47	62	11.55	
**Renal disease comorbidities**					
**Present**	63	14	29	22.22	0.304
**Absent**	1,230	209	317	16.99	
**Hepatic disease comorbidities**					
**Present**	325	74	118	22.77	0.003
**Absent**	968	149	228	15.39	
**Presence of allergy**					
**Present**	268	71	107	26.49	<0.001
**Absent**	866	133	209	15.36	
**Duration of treatment with raltegravir**					
**<90 days**	63	17	25	26.98	0.307
**90‒<180 days**	40	8	12	20.00	
**180‒<270 days**	56	9	16	16.07	
**270‒<1 year**	43	3	3	6.98	
**1‒<2 years**	196	20	25	10.20	
**2‒<3 years**	229	37	56	16.16	
**3‒<4 years**	248	40	65	16.13	
**4‒<5 years**	199	44	70	22.11	
**5‒<6 years**	141	26	42	18.44	
**≥6 years**	76	18	30	23.68	
**Concomitant medications**					
**Yes**	1,292	223	346	17.26	1.000
**No**	1	0	0	0.00	
**Number of concomitant medications**					
**1**	440	30	36	6.82	<0.001
**2**	199	24	32	12.06	
**3**	136	18	26	13.24	
**4**	105	20	22	19.05	
**≥5**	412	131	230	31.80	

^a^ *p*-values for Fisher’s exact test or Cochran-Armitage test

ADR: adverse drug reactions

### Analysis for effectiveness parameters

#### Virologic responses

**Fig 2:**
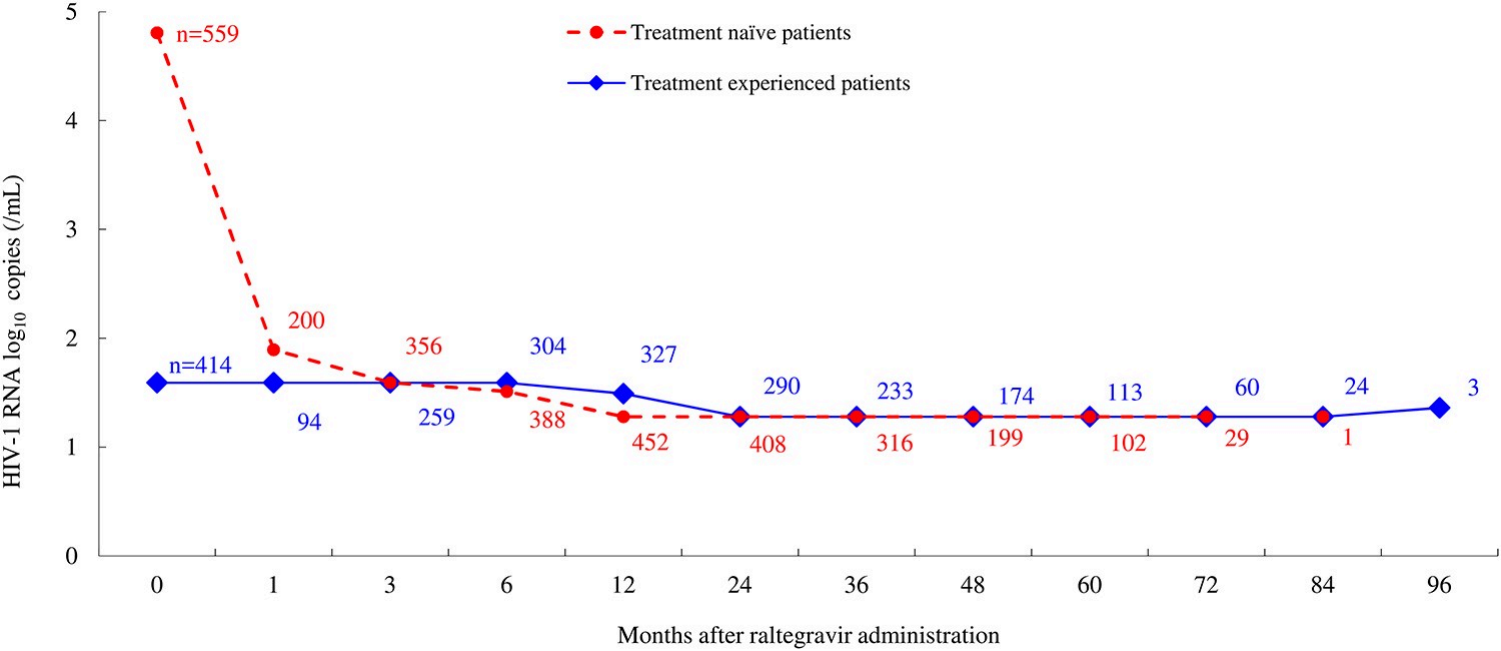
Changes in the median HIV-1 RNA viral loads of treatment-naïve patients and treatment-experienced patients. The markers indicate the median.

**Fig 3:**
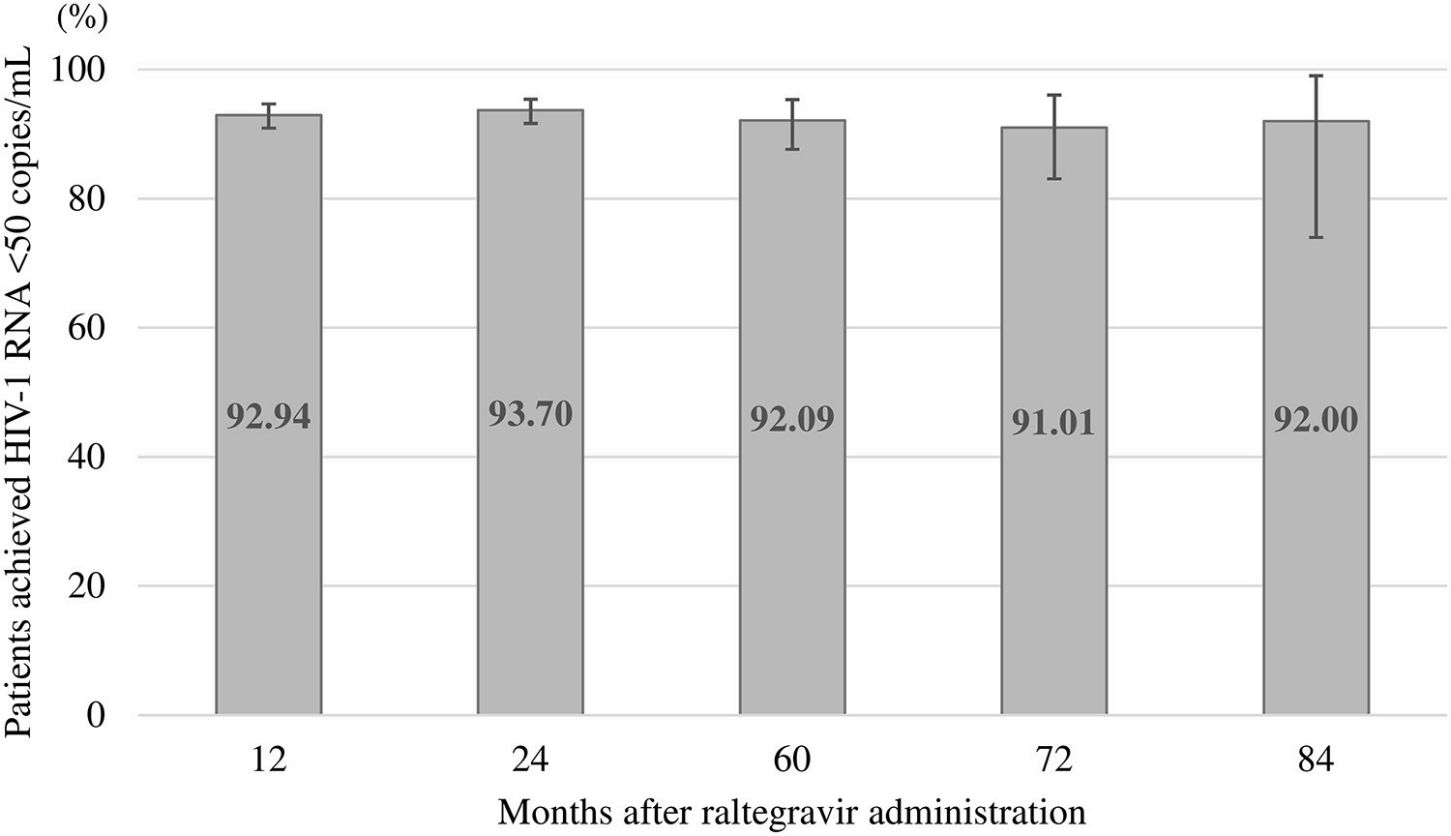
Proportion of patients with <50 copies/mL of HIV-1 RNA in patients treated with raltegravir. Error bars indicate 95% confidence intervals.

At the start of the treatment, the median HIV-1 RNA viral loads among the treatment-naïve patients were considerably higher compared with those of treatment-experienced patients (64,000 vs. 39 copies/mL), but this level rapidly decreased to 78.5 copies/mL after one month of raltegravir treatment. Median HIV-1 RNA viral loads in treatment-naïve and treatment-experienced groups each maintained below 40 copies/mL after three months of raltegravir treatment (Fig 2, S2 Appendix). Regarding long-term virologic suppression in both treatment-naïve and treatment-experienced patients, the proportion of patients with HIV-1 RNA viral loads of less than 50 copies/mL (including blip cases) was 81.79% (503/615 cases, 95%CI: 78.51–84.76) at 3 months and 89.47% (620/693, 86.94–91.65) at 6 months, and it exceeded 90% with 92.94% (724/779, 90.91–94.64), 93.70% (654/698, 91.63–95.38), 93.81% (515/549, 91.45–95.67), 93.30% (348/373, 90.26–95.62), 92.09% (198/215, 87.64–95.33), 91.01% (81/89, 83.05–96.04), and 92.00% (23/25, 73.97–99.02) at 12, 24, 36, 48, 60, 72, and 84 months, respectively. Although this proportion reached 100% (95%CI: 29.24–100.00) at 96 months, this group included only three treatment-experienced patients (Fig 3, S3 Appendix).

#### Immunologic responses

**Fig 4:**
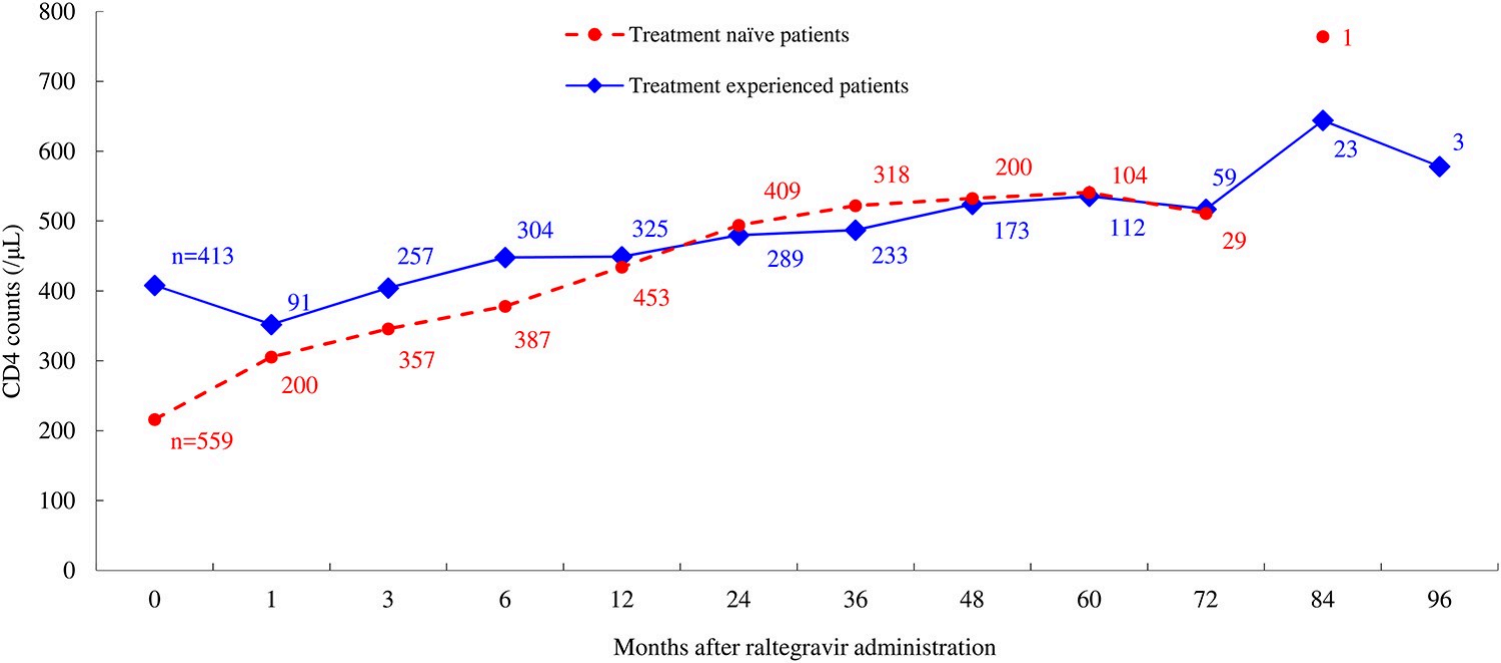
Change in median CD4+ cell counts of treatment-naïve patients and treatment-experienced patients. The markers indicate the median.

At the start of raltegravir treatment, the median CD4^+^ cell counts in treatment-naïve patients were lower compared with those in treatment-experienced patients (216 vs. 408 cells/μL), but the CD4^+^ cell counts in the treatment-naïve group reached 346 cells/μL after 3 months and 522 cells/μL after 36 months of raltegravir treatment (Fig 4, S4 Appendix). The CD4^+^ cell counts in both the treatment-naïve and treatment-experienced groups displayed an increasing trend throughout the treatment period; the CD4^+^ cell counts were maintained at over 500 cells/μL after 36 months in treatment-naïve patients and after 48 months in treatment-experienced patients.

#### CDC categories

Most of the patients assessed as belonging to category A (505/512 cases; 98.6%) and category B (51/53 cases; 96.2%) maintained their pre-treatment CDC category even after raltegravir administration. Only nine cases had disease progression.

## Discussion

This study was conducted using the results of a nine-year post-marketing surveillance on raltegravir in Japan. Data from 1,303 patients were initially extracted from the HRD cooperative survey, and, after excluding 10 ineligible patients, a total of 1,293 patients were included in the safety analysis. The overall ADR risk (17.25%) in this survey was fairly low, compared with previous international clinical trials reporting the ADR incidence for raltegravir, which ranged from approximately 44%‒61% with study periods spanning 1‒5 years [1–8]. Hyperlipidaemia and abnormal hepatic function were the most commonly reported ADRs in our study, with risks slightly exceeding 1%. No specific tendency was observed in the occurrence of other ADRs, including rash, cardiovascular disorders, elevated creatine kinase, and musculoskeletal and connective tissue disorders (i.e. rhabdomyolysis and myopathy) reported in previous clinical trials with a 2-year maximum of raltegravir administration [1,2,4,17]. Muscle symptoms including myalgia, pain in extremity and rhabdomyolysis (0.31%, 4 cases) and CPK increase (0.15%, 2 cases) were fairly low in this survey, compared with other observational study (SCOLTA project) in patients receiving raltegravir, which were 5.2% and 21.1%, respectively [18]. SCOLTA project also investigated drug-related central nervous system (CNS) symptoms which were 10.4%, whereas all psychiatric and nervous system disorders in this survey were low at 2.01% (26 cases) [19]. As for neoplasms, either benign or malignant, Kaposi’s sarcoma could be considered a disease-specific complication, but the proportion of all remaining malignancies was as low as 0.08%. For long-term safety, no specific tendency was observed in the overall ADR risk by raltegravir treatment period, but the risk of metabolism and nutrition disorders increased as the raltegravir treatment period lengthened. However, nearly 40% of the patients were 45 years of age or older at the initiation of raltegravir and many patients have comorbidities including diabetes mellitus or hyperlipidaemia, therefore it cannot be concluded that the trend was caused by long-term use of raltegravir. Although the risk of malignancies in patients treated with raltegravir should continue to be monitored, the <1% risk of most reported ADRs in this long-term safety assessment of a large Japanese cohort with over 1,000 patients suggests the general tolerability of raltegravir.

The effectiveness analysis was conducted on data from a total of 1,178 patients. The median HIV-1 RNA viral loads in treatment-naïve patients decreased rapidly after one month of raltegravir treatment and were identical in both treatment-naïve and treatment-experienced patients after 3 months of raltegravir treatment. Although decreasing numbers on raltegravir treatment as time goes on, using this as denominator, the proportion of patients with <50 copies/mL of HIV-1 RNA remained at 92.00% (23/25 cases, 95%CI: 73.97–99.02) in patients treated for 7 years, which is higher than or at a similar level as the corresponding proportions reported by previous clinical trials conducted overseas for a maximum of 5 years [1–8], suggesting a long-term virologic suppression by raltegravir treatment in Japanese patients.

Similarly, the median CD4^+^ cell counts in treatment-naïve patients were initially lower than those in treatment-experienced patients, but they rapidly increased to 346 cells/μL after 3 months of raltegravir treatment and were maintained at >500 cells/μL after 3 years of raltegravir in treatment-naïve patients and after 4 years in treatment-experienced patients. Previous studies reported that the changes in CD4^+^ cell counts from baseline were 189 cells/μL, 240 cells/μL, and 374 cells/μL in treatment-naïve patients (STARTMRK study) and 109 cells/μL, 123 cells/μL, and 293 cells/μL in patients with triple-class drug resistance in whom previous ART had failed (BENCHMRK-1 and -2 study) after approximately 1 year, 2 years, and 5 years of raltegravir administration, respectively [3–8]. In the present survey, we found an increase by 218 cells/μL, 278 cells/μL, and 325 cells/μL in treatment-naïve patients and by 41 cells/μL, 72 cells/μL, and 127.5 cells/μL in treatment-experienced patients after 1-, 2-, and 5-year treatment with raltegravir, respectively; this result may indicate an equivalent or better improvement regarding CD4^+^ cell counts resulted from virological suppression in raltegravir-treated Japanese patients. The observed improvement in the immunologic response even for treatment-experienced patients implies that an improvement for patients is likely after changing their ART regimen to include raltegravir.

There are several limitations to the present study. First, our results may be specific to particular groups of patients with HIV infection. The included patients in this study were registered at designated institutions, and, although in principle all patients receiving raltegravir were registered, each enrolment decision was made by contractor investigators. Therefore, a selection bias is inevitable. Second, the effectiveness results in this study cannot be purely attributed to raltegravir because antiretrovirals are commonly used in combinations. The present results support the effectiveness of raltegravir, but they must be interpreted with care. Third, as this data was drawn from HRD cooperative survey conducted by all companies having an HIV drug approved in Japan, we can only extract data of patients still on treatment with raltegravir at the time of the annual report. If patients collected as raltegravir data and switched for viral loads ≥50 copies/mL judged from collected effectiveness data, these patients were counted as failure. However, after they have switched to another ART regimen, we might not have been able to follow them any longer in this study. Therefore, a selection bias for over estimating effectiveness is inevitable. Fourth, as we had no control group for comparison, the safety and effectiveness results for raltegravir cannot be compared with those in HIV-infected patients who received no or other antiretroviral treatments. Last, data on patients who were treated with raltegravir for longer than 7 years were scarce, which may have limited the precision of those results.

In conclusion, long-term treatment with raltegravir is well-tolerated and effective regarding viral suppression, as measured by HIV-1 RNA levels and CD4^+^ cell counts, in Japanese HIV patients. Such benefits can be expected not only for patients who are about to start antiretroviral therapy but also for patients who switch antiretroviral regimens to one including raltegravir.

## Supporting information

S1 Appendix Adverse drug reactions and laboratory abnormalities reported in patients (n = 1,293). (DOCX)

S2 Appendix Changes in the median HIV-1 RNA viral loads of treatment-naïve patients and treatment-experienced patients. Detection threshold of HIV-1 RNA viral loads were treated as 39 until 2011, and 19 since 2012.
(DOCX)

S3 Appendix Proportion of patients with <50 copies/mL of HIV-1 RNA in patients treated with raltegravir. ^a^Case counts include blip cases (<50 copies/mL with transient increases up to 1000 copies/mL).
(DOCX)

S4 Appendix Change in median CD4+ cell counts of treatment-naïve patients and treatment-experienced patients. (DOCX)

## References

[1] Markowitz M, Nguyen BY, Gotuzzo E, Mendo F, Ratanasuwan W, Kovacs C, et al Protocol 004 Part II Study team. Sustained antiretroviral effect of raltegravir after 96 weeks of combination therapy in treatment-naïve patients with HIV-1 infection. J Acquir Immune Defic Syndr. 2009;52(3): 350–356. 10.1097/QAI.0b013e3181b064b0 19648823

[2] Gatell JM, Katlama C, Grinsztejn B, Eron JJ, Lazzarin A, Vittecoq D, et al Long-term efficacy and safety of the HIV integrase inhibitor raltegravir in patients with limited treatment options in a Phase II study. J Acquir Immune Defic Syndr. 2010;53(4): 456–463. 20306554 10.1097/qai.0b013e3181c9c967 PMC6075661

[3] Steigbigel RT, Cooper DA, Kumar PN, Eron JE, Schechter M, Markowitz M, et al Raltegravir with optimized background therapy for resistant HIV-1 infection. N Engl J Med. 2008;359(4): 339–354. 10.1056/NEJMoa0708975 18650512

[4] Steigbigel RT, Cooper DA, Teppler H, Eron JJ, Gatell JM, Kumar PN, et al Long-term efficacy and safety of raltegravir combined with optimized background therapy in treatment-experienced patients with drug-resistant HIV infection: week 96 results of the BENCHMRK 1 and 2 Phase III trials. Clin Infect Dis. 2010;50(4): 605–612. 10.1086/650002 20085491 PMC6076431

[5] Eron JJ, Cooper DA, Steigbigel RT, Clotet B, Gatell JM, Kumar PN, et al Efficacy and safety of raltegravir for treatment of HIV for 5 years in the BENCHMRK studies: final results of two randomised, placebo-controlled trials. Lancet Infect Dis. 2013;13(7): 587–596. 10.1016/S1473-3099(13)70093-8 23664333 PMC6083850

[6] Lennox JL, DeJesus E, Lazzarin A, Pollard RB, Madruga JV, Berger DS, et al STARTMRK investigators. Safety and efficacy of raltegravir-based versus efavirenz-based combination therapy in treatment-naïve patients with HIV-1 infection: a multicentre, double-blind randomised controlled trial. Lancet. 2009;374(9692): 796–806. 10.1016/S0140-6736(09)60918-1 19647866

[7] Lennox JL, Dejesus E, Berger DS, Lazzarin A, Pollard RB, Ramalho Madruga JV, et al Raltegravir versus efavirenz regimens in treatment-naive HIV-1-infected patients: 96-week efficacy, durability, subgroup, safety, and metabolic analyses. J Acquir Immune Defic Syndr. 2010;55(1): 39–48. 10.1097/QAI.0b013e3181da1287 20404738 PMC6065510

[8] Rockstroh JK, DeJesus E, Lennox JL, Yazdanpanah Y, Saag MS, Wan H, et al Durable efficacy and safety of raltegravir versus efavirenz when combined with tenofovir/emtricitabine in treatment-naïve HIV-1-infected patients: final 5-year results from STARTMRK. J Acquir Immune Defic Syndr. 2013;63(1): 77–85. 10.1097/QAI.0b013e31828ace69 23412015

[9] Merck & Co., Inc. Merck receives FDA Approval of ISENTRESS HD (raltegravir), a new once-daily option, in combination with other antiretroviral agents, for the treatment of HIV-1 infection in appropriate patients. http://investors.merck.com/news/press-release-details/2017/Merck-Receives-FDA-Approval-of-ISENTRESS-HD-raltegravir-a-New-Once-Daily-Option-in-Combination-with-Other-Antiretroviral-Agents-for-the-Treatment-of-HIV-1-Infection-in-Appropriate-Patients/default.aspx.

[10] Annual Report on AIDS Trends 2016, AIDS Surveillance Committee, Ministry of Health, Labour and Welfare. http://api-net.jfap.or.jp/status/2016/16nenpo/16nenpo_menu.html

[11] HIV-related drug co-operative survey. HRD website. https://www.hrd.gr.jp/index.html.

[12] Reliquet V, Allavena C, Morineau-Le Houssine P, Mounoury O, Raffi F. Twelve-year experience of nevirapine use: benefits and convenience for long-term management in a French cohort of HIV-1-infected patients. HIV Clin Trials. 2010;11(2): 110–117. 10.1310/hct1102-110 20542847

[13] Laurent C, Tchatchueng Mbougua JB, Ngom Guèye NF, Etard JF, Diouf A, Landman R, et al Long-term effectiveness and safety of didanosine combined with lamivudine and efavirenz or nevirapine in antiretroviral-naive patients: a 9-year cohort study in Senegal. Trop Med Int Health. 2011:16(2): 217–222. 10.1111/j.1365-3156.2010.02690.x 21087377

[14] Castelnuovo B, Kiragga A, Mubiru F, Kambugu A, Kamya M, Reynolds SJ. First-line antiretroviral therapy durability in a 10-year cohort of naïve adults started on treatment in Uganda. J Int AIDS Soc. 2016;19(1): 20773 10.7448/IAS.19.1.20773 27319742 PMC4913145

[15] Murphy RL, da Silva BA, Hicks CB, Eron JJ, Gulick RM, Thompson MA, et al Seven-year efficacy of a lopinavir/ritonavir-based regimen in antiretroviral-naïve HIV-1-infected patients. HIV Clin Trials. 2008;9(1): 1–10. 10.1310/hct0901-1 18215977

[16] Centers for Disease Control and Prevention. 1993 revised classification system for HIV infection and expanded surveillance case definition for AIDS among adolescents and adults. MMWR Recomm Rep. 1992:41; 1–19. 1361652

[17] Assessment Report for Isentress tablet 400 mg. http://www.pmda.go.jp/drugs/2008/P200800025/63015300_22000AMX01647_A100_1.pdf.

[18] Madeddu G, De Socio GV, Ricci E, Quirino T, Orofino G, Carenzi L, et al Muscle symptoms and creatine phosphokinase elevations in patients receiving raltegravir in clinical practice: Results from the SCOLTA project long-term surveillance. Int J Antimicrob Agents. 2015;45(3):289–294. 10.1016/j.ijantimicag.2014.10.013 25476452

[19] Madeddu G, Menzaghi B, Ricci E, Carenzi L, Martinelli C, di Biagio A, et al Raltegravir central nervous system tolerability in clinical practice: results from a multicenter observational study. AIDS. 2012;26(18):2412–2415. 10.1097/QAD.0b013e32835aa141 23032413

