# Supplementary material for: Long-term tolerability and effectiveness of raltegravir in Japanese patients: Results from post-marketing surveillance
Source: PLoS One. 2019 Jan 9;14(1):e0210384. doi: 10.1371/journal.pone.0210384 (PMC6326570; doi:10.1371/journal.pone.0210384)
Supplement: S1 Appendix — (DOCX) [file pone.0210384.s001.docx]

**S1 Appendix. Adverse drug reactions and laboratory abnormalities reported in patients (n = 1,293)**

| **Adverse drug reactions**  **(MedDRA system organ class and term)** | **Number of event** | | **(%)** |
| --- | --- | --- | --- |
| Infections and infestations (n = 11, 0.85%) |  | |  |
| Folliculitis | 1 | | (0.08) |
| Hepatitis C | 1 | | (0.08) |
| Herpes zoster | 3 | | (0.23) |
| Progressive multifocal leukoencephalopathy | 1 | | (0.08) |
| Viral upper respiratory tract infection | 1 | | (0.08) |
| Cytomegalovirus chorioretinitis | 1 | | (0.08) |
| Pneumocystis jirovecii pneumonia | 3 | | (0.23) |
| Neoplasms benign, malignant and unspecified  (including cysts and polyps) (n = 6, 0.46%) |  | |  |
| Bladder cancer | 1 | | (0.08) |
| Kaposi's sarcoma | 2 | | (0.15) |
| Skin papilloma | 1 | | (0.08) |
| Squamous cell carcinoma of lung | 1 | | (0.08) |
| Thymoma | 1 | | (0.08) |
| Blood and lymphatic system disorders (n = 6, 0.46%) |  | |  |
| Anaemia | 2 | | (0.15) |
| Anaemia macrocytic | 1 | | (0.08) |
| Iron deficiency anaemia | 1 | | (0.08) |
| Lymphadenopathy | 1 | | (0.08) |
| Thrombocytopenia | 1 | | (0.08) |
| Immune system disorders (n = 8, 0.62%) |  | |  |
| Immune reconstitution inflammatory syndrome | 8 | | (0.62) |
| Endocrine disorders (n = 1, 0.08%) |  | |  |
| Hypothyroidism | 1 | | (0.08) |
| Metabolism and nutrition disorders (n = 53, 4.10%) |  | |  |
| Dehydration | 1 | | (0.08) |
| Diabetes mellitus | 5 | | (0.39) |
| Hypercholesterolaemia | 4 | | (0.31) |
| Hyperglycaemia | 1 | | (0.08) |
| Hyperkalaemia | 1 | | (0.08) |
| Hypertriglyceridaemia | 8 | | (0.62) |
| Hyperuricaemia | 11 | | (0.85) |
| Obesity | 1 | | (0.08) |
| Dyslipidaemia | 9 | | (0.70) |
| Hyperphosphatasaemia | 3 | | (0.23) |
| Lipid metabolism disorder | 1 | | (0.08) |
| Hyperlipidaemia | 14 | | (1.08) |
| Type 2 diabetes mellitus | 1 | | (0.08) |
| Psychiatric disorders (n = 13, 1.01%) |  | |  |
| Anxiety | 1 | | (0.08) |
| Completed suicide | 1 | | (0.08) |
| Depression | 4 | | (0.31) |
| Hallucination, auditory | 1 | | (0.08) |
| Insomnia | 7 | | (0.54) |
| Irritability | 1 | | (0.08) |
| Nervous system disorders (n = 13, 1.01%) |  | |  |
| Dizziness | 2 | | (0.15) |
| Dizziness postural | 1 | | (0.08) |
| Facial paralysis | 1 | | (0.08) |
| Headache | 5 | | (0.39) |
| Hypoaesthesia | 1 | | (0.08) |
| Neuralgia | 1 | | (0.08) |
| Transient ischaemic attack | 1 | | (0.08) |
| Tremor | 1 | | (0.08) |
| Cognitive disorder | 1 | | (0.08) |
| Restless legs syndrome | 1 | | (0.08) |
| Eye disorders (n = 1, 0.08%) | 1 | | (0.08) |
| Eyelid oedema | 1 | | (0.08) |
| Cardiac disorders (n = 6, 0.46%) |  | |  |
| Atrial fibrillation | 1 | | (0.08) |
| Cardiac failure | 2 | | (0.15) |
| Myocardial infarction | 1 | | (0.08) |
| Supraventricular extrasystoles | 1 | | (0.08) |
| Ventricular extrasystoles | 1 | | (0.08) |
| Vascular disorders (n = 11, 0.85%) | 11 | | (0.85) |
| Hypertension | 9 | | (0.70) |
| Lymphoedema | 1 | | (0.08) |
| Peripheral arterial occlusive disease | 1 | | (0.08) |
| Respiratory, thoracic and mediastinal disorders (n = 4, 0.31%) |  | |  |
| Dyspnoea | 1 | | (0.08) |
| Rhinitis allergic | 2 | | (0.15) |
| Laryngeal haemorrhage | 1 | | (0.08) |
| Gastrointestinal disorders (n = 25, 1.93%) |  | |  |
| Abdominal discomfort | 2 | | (0.15) |
| Abdominal distension | 3 | | (0.23) |
| Abdominal pain | 1 | | (0.08) |
| Chronic gastritis | 3 | | (0.23) |
| Colitis ulcerative | 1 | | (0.08) |
| Constipation | 1 | | (0.08) |
| Diarrhoea | 2 | | (0.15) |
| Dyspepsia | 1 | | (0.08) |
| Gastric ulcer | 1 | | (0.08) |
| Gastrooesophageal reflux disease | 3 | | (0.23) |
| Intestinal perforation | 1 | | (0.08) |
| Irritable bowel syndrome | 1 | | (0.08) |
| Nausea | 3 | | (0.23) |
| Pancreatic pseudocyst | 1 | | (0.08) |
| Pancreatitis acute | 1 | | (0.08) |
| Small intestinal haemorrhage | 1 | | (0.08) |
| Haemorrhoidal haemorrhage | 1 | | (0.08) |
| Abdominal symptom | 1 | | (0.08) |
| Faeces soft | 1 | | (0.08) |
| Hepatobiliary disorders (n = 36, 2.78%) | |  |  |
| Hepatic function abnormal | | 17 | (1.31) |
| Hepatic steatosis | | 4 | (0.31) |
| Hepatocellular injury | | 1 | (0.08) |
| Hyperbilirubinaemia | | 2 | (0.15) |
| Liver disorder | | 11 | (0.85) |
| Drug-induced liver injury | | 1 | (0.08) |
| Skin and subcutaneous tissue disorders (n = 19, 1.47%) | |  |  |
| Alopecia | | 1 | (0.08) |
| Dermal cyst | | 1 | (0.08) |
| Dermatitis allergic | | 2 | (0.15) |
| Dermatitis atopic | | 1 | (0.08) |
| Drug eruption | | 2 | (0.15) |
| Haemorrhage subcutaneous | | 2 | (0.15) |
| Pruritus | | 4 | (0.31) |
| Rash | | 3 | (0.23) |
| Seborrhoeic dermatitis | | 1 | (0.08) |
| Urticaria chronic | | 2 | (0.15) |
| Eosinophilic pustular folliculitis | | 1 | (0.08) |
| Musculoskeletal and connective tissue disorders (n = 11, 0.85%) | |  |  |
| Back pain | | 3 | (0.23) |
| Myalgia | | 1 | (0.08) |
| Osteonecrosis | | 1 | (0.08) |
| Osteoporosis | | 2 | (0.15) |
| Pain in extremity | | 1 | (0.08) |
| Rhabdomyolysis | | 2 | (0.15) |
| Tenosynovitis | | 1 | (0.08) |
| Renal and urinary disorders (n = 19, 1.47%) | |  |  |
| Glomerulonephritis membranous | | 1 | (0.08) |
| Nephrolithiasis | | 1 | (0.08) |
| Nephropathy | | 1 | (0.08) |
| Renal disorder | | 2 | (0.15) |
| Renal failure | | 1 | (0.08) |
| Renal tubular necrosis | | 1 | (0.08) |
| Renal tubular dysfunction | | 1 | (0.08) |
| Renal impairment | | 11 | (0.85) |
| Acute kidney injury | | 2 | (0.15) |
| Prerenal failure | | 1 | (0.08) |
| Reproductive system and breast disorders (n = 1, 0.08%) | |  |  |
| Haematospermia | | 1 | (0.08) |
| Prostatitis | | 1 | (0.08) |
| General disorders and administration site conditions (n = 8, 0.62%) | |  |  |
| Death | | 4 | (0.31) |
| Feeling cold | | 1 | (0.08) |
| Malaise | | 1 | (0.08) |
| Pain | | 2 | (0.15) |
| Drug resistance | | 1 | (0.08) |
| Investigations (n = 55, 4.25%) | |  |  |
| Alanine aminotransferase increased | | 3 | (0.23) |
| Aspartate aminotransferase increased | | 1 | (0.08) |
| Beta 2 microglobulin urine increased | | 1 | (0.08) |
| Blood bilirubin increased | | 2 | (0.15) |
| Blood bilirubin unconjugated increased | | 1 | (0.08) |
| Blood cholinesterase increased | | 3 | (0.23) |
| Blood creatine phosphokinase increased | | 2 | (0.15) |
| Blood creatinine increased | | 7 | (0.54) |
| Blood lactate dehydrogenase increased | | 1 | (0.08) |
| Blood triglycerides increased | | 4 | (0.31) |
| Blood uric acid increased | | 4 | (0.31) |
| C-reactive protein increased | | 2 | (0.15) |
| Gamma-glutamyltransferase increased | | 5 | (0.39) |
| Blood urine present | | 1 | (0.08) |
| Haemoglobin decreased | | 1 | (0.08) |
| Lipids increased | | 1 | (0.08) |
| Liver function test abnormal | | 3 | (0.23) |
| Lymphocyte count decreased | | 1 | (0.08) |
| Platelet count decreased | | 1 | (0.08) |
| Weight increased | | 1 | (0.08) |
| White blood cell count increased | | 2 | (0.15) |
| Bone density decreased | | 4 | (0.31) |
| Protein urine present | | 2 | (0.15) |
| Transaminases increased | | 2 | (0.15) |
| Cystatin C increased | | 1 | (0.08) |
| Blood alkaline phosphatase increased | | 2 | (0.15) |
| Hepatic enzyme increased | | 2 | (0.15) |
| Renal function test abnormal | | 5 | (0.39) |
| Liver function test increased | | 4 | (0.31) |
| Injury, poisoning and procedural complications (n = 4, 0.31%) | |  |  |
| Fracture | | 1 | (0.08) |
| Intentional overdose | | 1 | (0.08) |
| Spinal compression fracture | | 1 | (0.08) |
| Stress fracture | | 1 | (0.08) |
