# Supplementary material for: Long-term tolerability and effectiveness of raltegravir in Japanese patients: Results from post-marketing surveillance
Source: PLoS One. 2019 Jan 9;14(1):e0210384. doi: 10.1371/journal.pone.0210384 (PMC6326570; doi:10.1371/journal.pone.0210384)
Supplement: S2 Appendix — Detection threshold of HIV-1 RNA viral loads were treated as 39 until 2011, and 19 since 2012. (DOCX) [file pone.0210384.s002.docx]

**S2 Appendix. Changes in the median HIV-1 RNA viral loads of treatment-naïve patients and treatment-experienced patients**

| HIV-1 RNA copies/mL | 0 m | 1 m | 3 m | 6 m | 12 m | 24 m | 36 m | 48 m | 60 m | 72 m | 84 m | 96 m |
| --- | --- | --- | --- | --- | --- | --- | --- | --- | --- | --- | --- | --- |
| Treatment naïve |  |  |  |  |  |  |  |  |  |  |  |  |
| Patients, n | 559 | 200 | 356 | 388 | 452 | 408 | 316 | 199 | 102 | 29 | 1 | - |
| Median | 64000 | 78.5 | 39 | 32.5 | 19 | 19 | 19 | 19 | 19 | 19 | 19 | - |
| Min | 19 | 11 | 19 | 19 | 19 | 19 | 19 | 19 | 19 | 19 | 19 | - |
| Max | 10000000 | 269000 | 1140000 | 2300 | 960000 | 8640 | 671 | 1070 | 290 | 39 | 19 | - |
| Treatment experienced |  |  |  |  |  |  |  |  |  |  |  |  |
| Patients, n | 414 | 94 | 259 | 304 | 327 | 290 | 233 | 174 | 113 | 60 | 24 | 3 |
| Median | 39 | 39 | 39 | 39 | 31 | 19 | 19 | 19 | 19 | 19 | 19 | 23 |
| Min | 19 | 19 | 19 | 19 | 19 | 19 | 19 | 19 | 19 | 19 | 19 | 19 |
| Max | 9300000 | 290000 | 167000 | 6600 | 180000 | 340000 | 25000 | 44000 | 970 | 160 | 160 | 55 |

Detection threshold of HIV-1 RNA viral loads were treated as 39 until 2011, and 19 since 2012.
