# Supplementary material for: Long-term tolerability and effectiveness of raltegravir in Japanese patients: Results from post-marketing surveillance
Source: PLoS One. 2019 Jan 9;14(1):e0210384. doi: 10.1371/journal.pone.0210384 (PMC6326570; doi:10.1371/journal.pone.0210384)
Supplement: S3 Appendix — aCase counts include blip cases (<50 copies/mL with transient increases up to 1000 copies/mL). (DOCX) [file pone.0210384.s003.docx]

**S3 Appendix. Proportion of patients with <50 copies/mL of HIV-1 RNA in patients treated with raltegravir**

|  | 1 m | 3 m | 6 m | 12 m | 24 m | 36 m | 48 m | 60 m | 72 m | 84 m | 96 m |
| --- | --- | --- | --- | --- | --- | --- | --- | --- | --- | --- | --- |
| Total patients, n | 294 | 615 | 693 | 779 | 698 | 549 | 373 | 215 | 89 | 25 | 3 |
| Patients with  ≥50 copies/mL, n | 147 | 104 | 55 | 23 | 10 | 4 | 3 | 2 | 0 | 1 | 0 |
| Rebounder | 1 | 8 | 18 | 32 | 34 | 30 | 22 | 15 | 8 | 1 | 0 |
| Patients with  <50 copies/mL^a^, n | 146 | 503 | 620 | 724 | 654 | 515 | 348 | 198 | 81 | 23 | 3 |
| % | 49.66 | 81.79 | 89.47 | 92.94 | 93.70 | 93.81 | 93.30 | 92.09 | 91.01 | 92.00 | 100.00 |
| 95% confidential intervals (CI) | (43.80-55.52) | (78.51-84.76) | (86.94-91.65) | (90.91-94.64) | (91.63-95.38) | (91.45-95.67) | (90.26-95.62) | (87.64-95.33) | (83.05-96.04) | (73.97-99.02) | (29.24-100.00) |

^a^Case counts include blip cases (<50 copies/mL with transient increases up to 1000 copies/mL).
