# Supplementary material for: Long-term tolerability and effectiveness of raltegravir in Japanese patients: Results from post-marketing surveillance
Source: PLoS One. 2019 Jan 9;14(1):e0210384. doi: 10.1371/journal.pone.0210384 (PMC6326570; doi:10.1371/journal.pone.0210384)
Supplement: S4 Appendix — (DOCX) [file pone.0210384.s004.docx]

**S4 Appendix. Change in median CD4+ cell counts of treatment-naïve patients and treatment-experienced patients**

| CD4^+^ cell counts/μL | 0 m | 1 m | 3 m | 6 m | 12 m | 24 m | 36 m | 48 m | 60 m | 72 m | 84 m | 96 m |
| --- | --- | --- | --- | --- | --- | --- | --- | --- | --- | --- | --- | --- |
| Treatment naïve |  |  |  |  |  |  |  |  |  |  |  |  |
| Patients, n | 559 | 200 | 357 | 387 | 453 | 409 | 318 | 200 | 104 | 29 | 1 | - |
| Median | 216 | 305.5 | 346 | 378 | 434 | 494 | 522 | 532 | 541 | 511 | 764 | - |
| Min | 0 | 9 | 2 | 2 | 5 | 24 | 67 | 17 | 87 | 66 | 764 | - |
| Max | 903 | 1084 | 1208 | 867 | 1126 | 1101 | 1150 | 1393 | 1280 | 1587 | 764 |  |
| Treatment experienced |  |  |  |  |  |  |  |  |  |  |  |  |
| Patients, n | 413 | 91 | 257 | 304 | 325 | 289 | 233 | 173 | 112 | 59 | 23 | 3 |
| Median | 408 | 352 | 404 | 448 | 449 | 480 | 487 | 524 | 535.5 | 517 | 644 | 578 |
| Min | 0.1 | 2.9 | 0.3 | 3.2 | 14 | 45 | 53 | 79 | 124 | 88 | 139 | 390 |
| Max | 4810 | 976 | 1211 | 1446 | 1563 | 1459 | 1202 | 1623 | 1539 | 1949 | 1281 | 927 |
